# Delivering emoji/icon-based universal health education messages through smartphones

**DOI:** 10.3934/publichealth.2019.3.242

**Published:** 2019-07-22

**Authors:** Sudip Bhattacharya, Amarjeet Singh, Roy Rillera Marzo

**Affiliations:** 1 Department of Community Medicine, Himalayan Institute of Medical Sciences (HIMS), Swami Rama Himalayan University, Dehradun, India; 2 Department of Community Medicine, PGIMER, Chandigarh, India; 3 Deputy Dean, Asia Metropolitan University, Johor Bahru, Malaysia

**Keywords:** health communication, primary care, smartphones, health messages, public health, emojis, icons

## 1. Introduction

Health information technology (HIT) refers to the comprehensive management of health information across computerized systems and its secure exchange between patients, health care providers, government, and others [1],[2].

A broad and consistent use of HIT can potentially improve the quality of primary health care, reduce medical errors, reduce basic health care costs, decrease paperwork, increase administrative efficiency, and expand access to affordable care. The World Health Organization has identified HIT as a tool with which to address health problems and transform quality of life [3].

India is a hub of IT-enabled service industries. Despite this tremendous potential, there is limited use of HIT in the public health sector as compared with other sectors such as banking, railway, tourism, and entertainment, which have embraced IT on a considerable scale [1].

**Figure 1.**
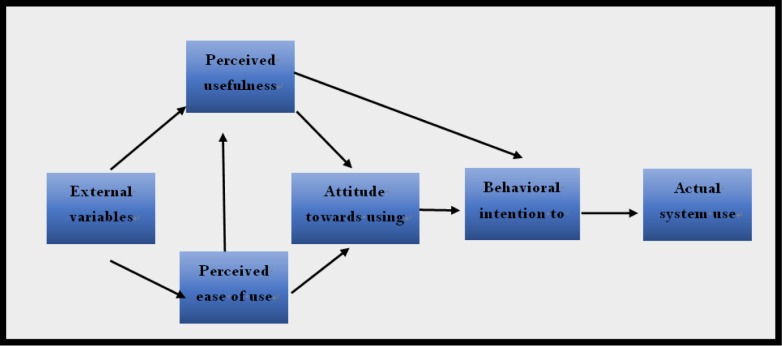
Technology acceptance model [4].

According to the technology acceptance model [4], the speed of acceptance and dissemination of a particular technology depends primarily on its perceived usefulness and ease of use (Figure 1). England et al. [5] conducted a study which revealed that the complexity of health care organizations and their fragmented internal structure constrained their ability to adopt organization-wide IT. The authors concluded that both organizational and technological factors lead to the slow adoption of strategic IT.

Another important contributing factor to the limited use of IT in health care is the comparatively less revenue generation in the public health sector. For most governments, primary health care is an investment with no immediate return though there are obvious long-term benefits. Such myopic perception at the policy level may function as a barrier to the adoption of any technology [6].

Other reasons of low use of technology in health sector include language barriers, use of outdated technology, and lackluster appeal for the general public [1].

With use of the smart phones by general public increasing day by day, an important way of use of HIT is popularization of emojis, images, and icons are pictorial methods of expression as health-related communication.

India is now considered a major mobile technology consumer [7], and communication through mobile phones is no longer limited to text messages. We communicate more with each other using emojis, images, and icons in chats or on social media.

**Figure 2.**
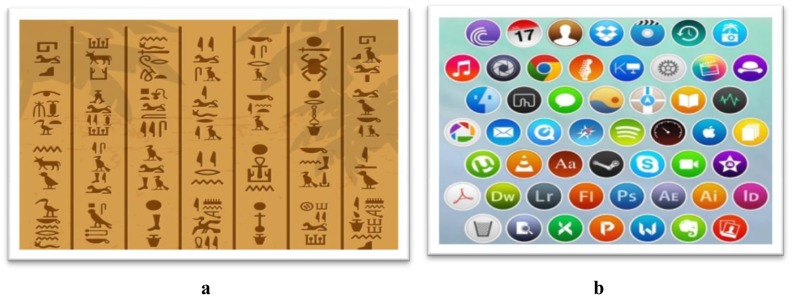
Similarity in symbolic communication earlier and now (Source-https://www.freepik.com/free-photos-vectors/design).

The inclination toward this pictorial mode of expression is quite natural. If we consider paleo-anthropology and the history of linguistic development, we can clearly see that Homo Sapiens have always used symbolic languages and wall paintings to communicate with each other (Figure 2). Even the earliest traces of language, dating back to the age of the caveman and woman, were also strikingly similar to icons, paintings, and symbols. These are still observed in tribal hamlets. The symbol/icon is thus a connecting link between our past and the present.

Emojis, images, and icons help break the barriers of languages. Meanings of symbols are easily understood across the globe. Furthermore, images tend to have more appeal than text. As the saying indicates, “A picture speaks more than a thousand words.” Interestingly, the written language (script) of some Asian countries (e.g., China, Korea, and Japan) are symbol-based. Apart from being linked with human emotions, Emojis, images, and icons can also be developed or tailor-made to appeal to the cultural norms prevalent in a society. One cannot ignore the strong influence of culture [6].

## 2. Use of symbols in health care and disease management

In Japan, a study was conducted on the use of graphic symbols to improve communication. Initially, graphic symbols were developed to correspond to 26 basic disease symptoms. It was observed that out of 26 symbols, 10 symbols showed > 90% matching rates for all groups. These symbols were considered to be effective alternatives to verbal communication [8].

We often use symbols in charts and pictures in primary health/public health during dietary assessment and in growth monitoring of children under 5 years of age. Symbols are also used in hospitals in the treatment of children with learning disorders, such as autism, dyslexia, and attention deficit hyperactivity disorder [8].

In patients receiving palliative care, or patients who cannot communicate, symbols are highly useful in the assessment of daily needs such as when the patient needs to take a drink, needs a toilet pan, or does not like a bitter medication [8]–[9].

Mobile phone technology is extensively used by the government to send primary health care-related information in the form of short message service messages (e.g. polio prevention). However, if the person/patient/target audience is illiterate or does not know the language in which these are sent, the entire exercise becomes fruitless. The practical significance of this method is, in effect, nullified by the language barrier.

Migration to different places for better employment and civic facilities is a natural consequence of the trend of rapid urbanization and socioeconomic transition that India is currently witnessing.

Consider, for example, a case where a person from Bengal province (who does not know the Punjabi language) is settled or working in Punjab province owing to various factors and is using a mobile SIM from Punjab. None of the public health/primary health care messages sent in the Punjabi language on a regular basis to the person through his or her mobile phone are understood. Though the health department provides health information through traditional methods (e.g. face-to-face communication, pamphlets, leaflets, banners), these methods are not always effective, especially in hard-to-reach areas or areas with significant cultural or regional differences [1].

According to the Linguistic Survey of India, the country has a total of 179 languages and 544 dialects. Health communication is a big challenge for primary health care workers, especially in hard-to-reach/tribal areas [10]. Previous studies have found that owing to communication barriers, tribal patients in India still prefer home delivery by untrained *dais* (women, who can conduct deliveries in their community), despite all efforts for institutional delivery by the government. The decisions of *Jan gurus* (local priests) regarding day-to-day health problems are final, which often bypasses primary/rural health care [11]–[16]. Clearly, cultural appropriateness is an indispensable quality of effective health care message delivery. In a country such as India, which is highly diverse, a deeper understanding of the rural community is important for effective public health message delivery [17].

Furthermore, an enormous amount of money is spent on printing leaflets, banners, sending short message services in different languages, etc. Yet the outcome assessment may not give very encouraging figures. Therefore, it is necessary to find alternative methods of providing health information.

Thus, a different strategy is required. The blanket approaches mentioned above [1] do not suffice. To make these efforts productive and rewarding, health messages should be delivered using a universal format understood by all recipients. These messages should also have better appeal and should capture the attention of the target audience.

## 3. Recommendations

We, therefore, propose that health messages may also be delivered through emojis, icons, and pictorial messages in addition to the traditional methods through smart phones. Smartphones can be employed in many ways in the delivery of health care services. For example, smartphones can be used to enhance the flow of information between primary health care providers and the general public, which in turn would provide more opportunities for health promotion and obtaining feedback.

**Figure 3.**
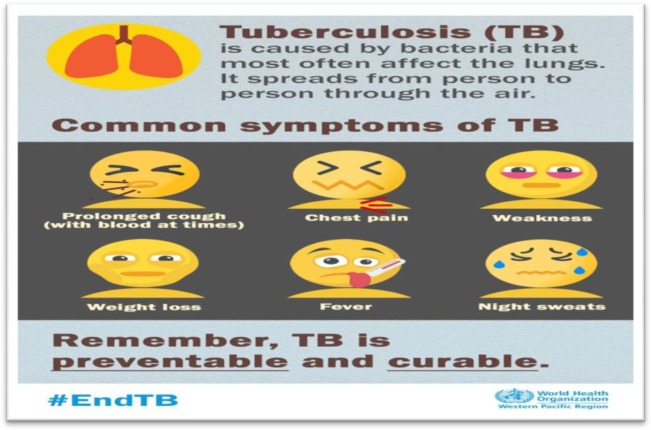
WHO is using symbols for delivering health message (Source-https://twitter.com/WHOPhilippines).

India is a diverse country with striking differences in culture, norms, and beliefs. In the 21st century, smart phone technology is increasingly used as a one-step solution for many of the problems of daily life. Emoji-based/symbolic health messages should be employed in primary health care. The World Health Organization recently began using emoji-based health messages for Tuberculosis patients (Figure 3). Most importantly, culture-specific icons/symbols need to be devised that may appeal to all, irrespective of language barriers, since these are related to our roots [1].

As various contextual factors contribute to symbol comprehension, more attention should be paid to describing the design of emojis, as well as considerations for developing them. There is also a need for symbols to be tested prior to use. These graphical symbols to be vetted prior to use applying the International Standard Organization's test method for comprehension.

In this way, effective health communication related to primary health can be made more easily available and palatable to the target/rural/marginalized/tribal population with good and sustained impact and at low cost.
